# Phytoplankton can bypass nutrient reductions in eutrophic coastal water bodies

**DOI:** 10.1007/s13280-017-0980-0

**Published:** 2017-11-21

**Authors:** Maximilian Berthold, Ulf Karsten, Mario von Weber, Alexander Bachor, Rhena Schumann

**Affiliations:** 1 Institute of Biological Sciences, Applied Ecology and Phycology, Biological Station Zingst, University of Rostock, Mühlenstraße 27, 18374 Zingst, Germany; 2 Institute of Biological Sciences, Applied Ecology and Phycology, University of Rostock, Albert-Einsteinstraße 3, 18051 Rostock, Germany; 3 State Agency for Environment, Nature Conservation and Geology Mecklenburg-Vorpommern, Goldberger Straße 12, 18273 Güstrow, Germany; 4 Oelmannsallee 5, 23909 Ratzeburg, Germany

**Keywords:** Chlorophyll a, Cyanobacteria, Eutrophication, Nutrient ratios, Precipitation

## Abstract

The EU-water framework directive aims at nutrient reductions, since anthropogenically induced eutrophication is a major threat for coastal waters. However, phytoplankton biomass in southern Baltic Sea coastal water bodies (CWB) remains high and the underlying mechanisms are not well understood. Therefore, a CWB data set was analysed regarding changes in phytoplankton biomass and nutrient concentration of nitrogen (N) and phosphorus (P) from 2000 to 2014. It was expected to find imbalances between produced phytoplankton biomass and total nutrient concentrations. Inner CWB were cyanobacteria-dominated and showed up to five times higher chlorophyll *a*-concentrations compared to outer CWB with similar total phosphorus-concentrations. Phytoplankton tended to be P-limited during spring and N-limited during summer. Phytoplankton biomass and nutrient concentrations were even higher during very humid years, which indicated a close coupling of the CWB with their catchment areas. This study suggests that re-mesotrophication efforts need to consider the importance of changed phytoplankton composition and nutrient availabilities.

## Introduction

Many coastal waters have deteriorated since the early 1950s by nutrient inputs from chemical fertilisers used in intensive agricultural practices. Nutrients are transported from the catchment area of a water body by erosion, direct discharge or groundwater transport (e.g. Magnien et al. 1992; Lillebø et al. 2012; Lemley et al. 2014). The overload of nutrients prevents nutrient limitations on phytoplankton and macrophytes, which then excessively grow producing extensive amounts of biomass. Eutrophication results in higher water turbidity, changed species composition of plankton and food webs as well as hypoxia or anoxia above sediments. Algal blooms often occur and reduce the provided ecosystem services (Nobre 2009), and the ecosystem function, for example, by mucilage or toxin production (Anderson et al. 2008).

There are many counteractions to reduce the impact of anthropogenic eutrophication (e.g. EU-Water Framework Directive—WFD, EU-Marine Strategy Framework Directive—MSFD, Clean Water Act, National Estuary Program, UN Oceans Compact). As for the European Union (EU), the WFD and MSFD aim at a “good ecological state” for all aquatic systems within the next years (by the year 2027 at the latest). The “good ecological state” is defined by abiotic (e.g. dissolved and total nutrients) and biotic parameters (e.g. species composition and chlorophyll *a* concentration) to set target values of those factors. The target value is calculated by comparison with a reference value that is derived from the time before human impact. Measures to reach the target value include, for example, changed hydrological processes (e.g. Flemer and Champ 2006), nutrient reductions from all terrestrial sources (e.g. Stigebrandt et al. 2014) or biomanipulation (e.g. van Keulen et al. 2003).

Improved water treatment plants lowered nutrient reductions from terrestrial sources. For the German coastal zones of the Baltic Sea, new water treatment plants in the early 1990s reduced their own direct inflow of phosphorus (P) by 98% during the last two decades (Nausch et al. 2011). However, Germany needs to reduce its P inflow further. The newly defined reduction values aim at preventing 170 t of P entering the coastal waters of the German Baltic coast (HELCOM 2013). P is assumed to be the limiting nutrient in limnetic systems, whereas it is supposed that nitrogen (N) limits primary producers in marine systems (see Elser et al. 2007, and sources cited therein). Shallow freshwater systems and estuarine water bodies suffer especially from anthropogenic eutrophication (Nixon 1995; Vidal et al. 1999), because they are often connected to rather large catchment areas. Enclosed coastal waters receive the whole nutrient load from the catchment (Livingston 2001) and typically cannot release this load into the open ocean since they ecologically act as nutrient filter. Nevertheless, the external reduction of P inflow improved the ecological state in some aquatic systems (e.g. Kemp et al. 2005). The possible restoration success by reducing a sole nutrient source is a controversial issue (e.g. Duarte et al. 2009; Riemann et al. 2016). One important aspect concerning nutrient manipulation is related to the question whether the trophy, i.e. the systems production, changes proportionally to the nutrient reduction. Duarte et al. (2009, 2015), for example, mentioned that aquatic systems themselves might show a hysteresis process. Such a hysteresis process shifts the baseline that is necessary to reach a certain trophic state, i.e. a much lower nutrient availability is needed to reduce phytoplankton biomass again compared to eutrophication inducing fluxes. Any recovery might be delayed due to the increased P availability in the catchment area, distinctive weather events, highly turbid water bodies and changed phytoplankton species composition.

Any possible changes in P conditions in the catchment area must thus be included in management efforts. The Baltic proper, for example, shows increasing total phosphorus (TP) concentrations, even after strong P reduction of numerous external point sources, due to internal sources (Stigebrandt et al. 2014). These authors highlighted the importance of internal nutrient supply, for example from sediments, because of anoxic conditions. However, such anoxic conditions do not exist in coastal waters of the German Baltic coast, because of regular wind-induced mixing. This mixing leads to high oxygen concentration above the sediment at most observed stations throughout the year (>6 mg O_2_ mg L^−1^) (Bachor et al. 2013). These findings exclude remobilisation of internal P sources and rather point to external P sources within the catchment area, for example, from agricultural soils. The accumulated P in agricultural soils (van Dijk et al. 2016) can be partly released after soil disturbance, such as storm or heavy rain events promoting erosion (Jordan et al. 2003). The mobilised P will be flushed into the coastal water bodies (CWB) and support algal production. Although CWB with direct contact to rivers receive higher nutrient loads during years with enhanced precipitation, those CWB without direct contact to a river can be influenced by the surrounding land. Zimmer et al. (2016) showed that high TP concentrations (up to 4.5 mmol L^−1^) could be found in tile drains entering river tributaries during storm events. Nutrient inputs into main rivers like the Recknitz and Barthe, which flow into the Darss Zingst Bodden Chain, were reduced by more than 80% during the last 25 years. However, these rivers also showed increased nutrient concentrations after storm events thereby transporting this extra load into the respective CWB (Bachor et al. 2013). These pulse-like nutrient supplies or permanent small nutrient leakages from the surrounding land may support phytoplankton species, especially those adapted to low nutrient concentrations.

Particularly planktonic cyanobacteria can be very resilient to nutrient depletion, because they can store P intracellularly as polyphosphates (Aubriot et al. 2011) or substitute P, for example, by sulphur in their cell membrane for further growth (Van Mooy et al. 2009). Cyanobacteria increase turbidity over-proportionally, when their relative biomass of the total phytoplankton composition and the total nitrogen: total phosphorus (TN:TP) ratio is high (Smith 1986). Enhanced turbidity suppresses other, mainly eukaryotic phytoplankton species (Scheffer et al. 1994). Therefore, these specific features of cyanobacteria have to be taken into account in water quality measures since they still ecologically benefit from reduced nutrient inputs by closing point sources.

The main hypothesis of this study was that the described external P reductions over the last decades are still not sufficient to control phytoplankton biomass production in CWB of the German Baltic Sea. These analyses included long-term data on total and dissolved N and P forms as well as precipitation events, and their effects on phytoplankton composition and biomass, and finally turbidity. Meso- and eutrophic sites at the outer German Baltic Sea coast were compared to eutrophic inner CWB. The Chlorophyll *a* (chl *a*) concentration as a proxy for phytoplankton biomass, the impact of species composition and N or P as the limiting nutrient were analysed in a 15-year data set, which originated from the regular monitoring programme of the environmental state agency. Such long-term data are important for any management policy for CWB not only of the Southern Baltic Sea.

## Materials and methods

### Area of investigation

**Fig. 1:**
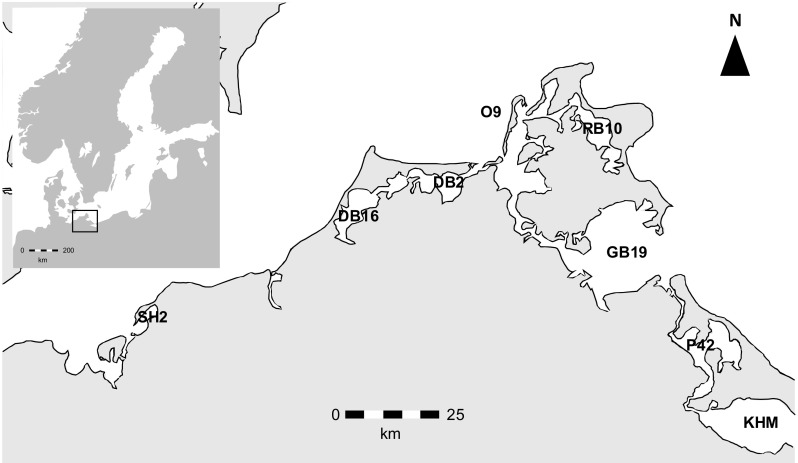
Map of the sampling area at the southern Baltic Sea Coast. Stations were named according to the permanent buoys where the samples were taken. Buoys were in the following coastal waters: SH2—Salzhaff, DB16—Saaler Bodden, DB2—Grabow, O9—Arkonasee, RB10—Großer Jasmunder Bodden, GB19—Greifswalder Bodden, P42—Peenestrom, KHM—Kleines Haff

Nutrients, biomass and phytoplankton data were analysed at eight stations of the Southern German Baltic Sea coast (Fig. 1). The station O9 (54°37,4′N, 13°01,7′E) is part of the outer coastline. The stations SH2 (54°03,8′N, 11°33,9′E), GB19 (54°12,4′N, 13°34,0′E) and KHM (53°49,5′N, 14°06,0′E) have direct contact with the Baltic Sea and are only semi-enclosed. The stations DB16 (54°20,1′N, 12°26,9′E), P42 (54°01,4′N, 13°45,6′E), DB2 (54°23,5′N, 12°50,3′E) and RB10 (54°30,4′N, 13°29,4′E) have at least one other coastal water body between them and the open Baltic Sea. The rivers Recknitz and Oder, respectively, directly influence the stations DB16 and KHM. Some general characteristics of the observed stations are shown in Table 1. More information regarding the coastal water zones of the German Baltic Sea coast can be found in Schiewer (2007). Sites were analysed in groups according to the chl *a* ranges reached in the respective systems.

**Table 1:** Main characteristics of the investigated coastal water bodies from west to east (except O9 as station of the open Baltic sea). SH2—Salzhaff, DB16—Saaler Bodden, DB2—Grabow, O9—Arkonasee, RB10—Großer Jasmunder Bodden, GB19—Greifswalder Bodden, P42—Peenestrom, KHM—Kleines Haff. Station DB2 and DB16 are part of the same lagoon and catchment area. Ratio of surface area to catchment area for KHM includes the whole Odra catchment area. Data for water depth, salinity, ratio surface to catchment, coastline and water exchange are from Schiewer (2007) and sources cited therein. Data for precipitation (2000–2014, as median) are from the German Meteorological Service (DWD) and Biological Station Zingst (BSZ)

	Mean depth (m)	Salinity	Average precipitation (mm a^−1^)	*A*_surface_:*A*_catchment_	Coastline: surface (km km^−2^)	Water exchange rate (a^−1^)
SH2	2.3	10.5	625	1:15	1.62	n.a.
DB16	2	1–3	610	1:8	0.93	0.15
DB2		8–10			0.65	
RB10	5.3	8–9	602	1:2	1.35	0.14
GB19	5.8	7.5	611	1:1.3	0.4	0.1
P42	n.a.	1–8	573	n.a.	0.73	n.a.
KHM	3.8	0.5–2	531	1:180	0.35	0.15

### Data set and sample treatment

The analysed data sets were derived from the State Agency for Environment, Nature Conservation and Geology Mecklenburg-Vorpommern (abbreviation in German: LUNG), the Biological Station Zingst (BSZ) and the German Meteorological Service (DWD). The LUNG data sets included total and dissolved N and P, phytoplankton, and secchi depth. Nutrient ratios were calculated from those data sets. All data were separated into the four seasonal periods of winter (December–February), spring (March–May), summer (June–August), and autumn (September–November).

The abiotic factors included total phosphorus (TP), dissolved inorganic phosphorus (DIP), total nitrogen (TN), dissolved inorganic nitrogen (DIN) and precipitation (as monthly and annual sum). DIN is the sum of nitrate, nitrite and ammonium. Samples for dissolved nutrients were stored dark until filtration through cellulose-acetate filters (pore size 0.45 µm) followed by freezing prior analysis. DIP was determined as soluble reactive phosphorus by using the molybdenum blue method (Strickland and Parsons 1972). Soluble reactive phosphorus means that the results can be overestimated due to hydrolysis of acid-labile P during the analysis. However, the percentage share of non-bioavailable DIP was not determined. Precipitation values were used from meteorological stations of the DWD and the BSZ closest to sampling stations. Extreme precipitation events refer to precipitation that exceeded the long-term median by at least 50% the amount (data not shown, source DWD). TP and TN were digested in a microwave with peroxodisulfate (DIN11905-1 1998). Total and dissolved nutrients were measured in a continuous flow analyser (Skalar Inc., later Alliance Instruments, (Malcolm-Lawes and Wong 1990; DIN13395 1996; DIN11905-1 1998; DIN11732 2005).

The biotic factors include biovolume of phytoplankton and chl *a* as the proxy for phytoplankton biomass. Phytoplankton samples were differentiated into 15 groups. However, only Chlorophyta, Cyanobacteria and Bacillariophyceae were further analysed because of their dominant occurrence in all samples. Phytoplankton biovolume was only available for the years 2007–2014. Bacillariophyceae were used as indicator for typical spring bloom situations and, hence, as important part of aquatic food webs. Chlorophyta and Cyanobacteria served both as indicators for eutrophicated water bodies. Species were taxonomically identified using light microscopy (Nikon inverted microscope, phase contrast, magnifications 200x and 400x, ECLIPSE Ti-S) according to HELCOM (2015b) (synonyms according to the most recent list of www.algaebase.org). Single cells of rod-shaped cyanobacteria and colony forming *Synechococcus/Cyanobium* clade cyanobacteria as well as Chroococcales were evaluated for all sampling sites in 2006–2010 (Schumann et al. 2008, 2012). The chl *a* concentration was determined photometrically after cold ethanol extraction overnight (HELCOM 2015a).

Precipitation values originated from meteorological stations of the DWD and the BSZ closest to sampling stations. Extreme events refer to years with high precipitation that exceeded the long-term median by at least 50% the amount (data not shown, source DWD).

### Statistical analysis

The statistical analysis was conducted with SigmaPlot 13.0. The Spearman Rank Order tested correlations between parameters. A *t* test was used to check on significant differences between years with average and extreme precipitation. Normality was tested by using a Shapiro–Wilk test. Results stated as significant had at least a significance level of *p* < 0.05.

## Results

### Chl *a* production as function of P and N availability

The DIP concentrations as long-term median were at 7 out of 8 stations below 0.6 µmol L^−1^ during winter time (Table 2). Further, the summer DIP concentrations were only at two stations (O9 and GB19) considerably lower (more than 50%), compared to winter. DIP concentrations that were at least twice the long-term median occurred more frequently during summer, compared to winter months at 6 out of 8 stations (summer: O9—15%, SH2—24%, GB19—26%, KHM—27%, P42—35%, DB16—44%). Only station KHM showed always detectable DIP concentration (>0.02 µmol L^−1^) during summer and winter. Interestingly, two of the innermost coastal waters (DB16 and RB10) exhibited always very low DIP concentrations, compared to the other stations.

**Table 2:** Concentrations of total nitrogen (TN), total phosphorus (TP), dissolved inorganic nitrogen (DIN) and dissolved inorganic phosphorus (DIP) in [µmol L^−1^] during the seasons spring (I; March, April, May), summer (II; June, July, August) and autumn months (III; September, October, November), and winter (IV; December, January, February), at the measured stations (Fig. 1). Results are presented as long-term median between 2000 and 2014. O9—Arkonasee, SH2—Salzhaff, DB2—Grabow, GB19—Greifswalder Bodden, RB10—Großer Jasmunder Bodden, KHM—Kleines Haff, P42—Peenestrom, DB16—Saaler Bodden

	TN				TP				DIN				DIP				TN:TP				DIN:DIP			
	I	II	III	IV	I	II	III	IV	I	II	III	IV	I	II	III	IV	I	II	III	IV	I	II	III	IV
O9	18	20	18	21	0.6	0.7	0.9	0.9	2.8	1	1.3	5.1	0.3	0.2	0.5	0.6	30	27	21	21	11	6	5	8
SH2	60	38	34	95	0.7	2.1	1	1.1	35.8	2.2	3.6	63.5	0.1	1	0.3	0.4	67	17	31	95	155	2	15	162
DB2	96	73	67	97	2.2	2.2	1.9	2.1	29.8	1.3	1.5	35.8	0.1	0.1	0.1	0.1	45	36	37	48	131	9	17	36
GB19	38	34	32	39	1	1.5	1.5	1.4	6.7	1	1.8	13.4	0.1	0.3	0.6	0.6	40	23	22	25	45	4	4	19
RB10	60	61	50	58	1.8	2.5	1.9	1.4	2.4	1.1	1.5	14.1	0.1	0.1	0.2	0.1	32	24	28	40	29	8	8	141
KHM	154	85	82	111	3.3	6.4	6.2	4.2	86	1.2	14.9	80	0.3	2.2	2.3	1.9	43	14	13	34	154	1	5	49
P42	121	105	100	99	2.3	4.8	3.8	2.4	41.7	0.8	4.1	55	0.1	0.5	0.5	0.6	46	19	25	43	202	3	11	122
DB16	207	154	164	203	4.0	4.1	3.6	4.3	57.2	1.7	1.9	62.1	0.1	0.1	0.1	0.1	48	40	47	47	234	9	16	454

DIN concentrations were at all stations highest in winter (5–80 µmol L^−1^) and lowest during summer (1–2 µmol L^−1^) (Table 2). The excess of DIN during winter and spring influenced the DIN:DIP ratio, which was above 16:1 at 7 out of 8 stations. However, the ratio dropped for all stations below 10:1 during summer, due to low DIN, but still determinable DIP concentrations (> 0.05 µmol L^−1^). The share of DIP to TP was lowest for all stations (2–14%) except O9 (50%) during spring, which occurred together with the lowest DIN:DIP and highest TN:TP ratios.

**Fig. 2:**
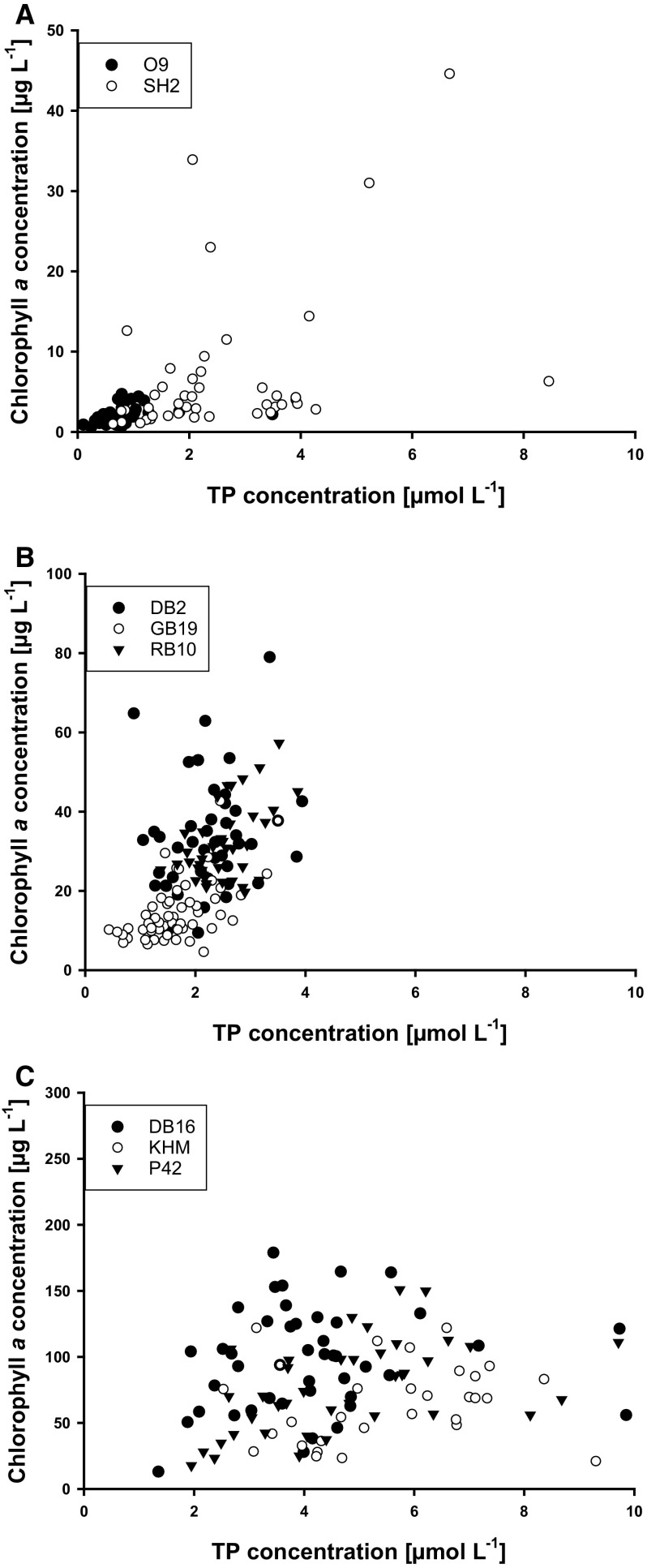
Total phosphorus (TP) concentrations (µmol L^−1^) plotted against the chlorophyll *a* (chl *a*) concentration (µg L^−1^) during the summer months (June, July, and August). The stations were sorted regarding their maximum concentrations for better overview. Please note the different scalings for chl *a* between **a** and **c**. Data set from the LUNG, O9 *n* = 36, SH2 *n* = 44, DB2 *n* = 44, GB19 *n* = 51, RB10 *n* = 40, DB16 *n* = 45, KHM *n* = 38, P42 *n* = 42. O9—Arkonasee, SH2—Salzhaff, DB2—Grabow, GB19—Greifswalder Bodden, RB10—Großer Jasmunder Bodden, KHM—Kleines Haff, P42—Peenestrom, DB16—Saaler Bodden

There was considerable variation between the amounts of TP and chl *a* during summer months (Fig. 2a–c). The stations SH2 and O9 showed the lowest chl *a* concentrations, even though SH2 is part of an inner coastal water Fig. 2a). TP and chl *a* correlated positively and significantly (O9, *r* = 0.597, *p* < 0.001, *n* = 36; SH2, *r* = 0.452, *p* = 0.002, *n* = 44). The long-term summer median TP concentrations were 0.7 and 2.1 µmol L^−1^ for O9 and SH2, respectively. The chl *a* concentrations amounted to 2 µg L^−1^ for O9 and 3.5 µg L^−1^ for SH2. The median Secchi depth was 4.8 m and 3.8 m, respectively. The outliers of chl *a* and TP occurred together with events of previous strong precipitation during the summer months of 2002, 2007, 2010 and 2011. The inner CWB of the stations DB2, GB19 and RB10 showed similar median TP concentrations as the station SH2 (DB2—2.2, GB19—1.6, RB10—2.5 µmol L^−1^). However, the amount of produced chl *a* was at least six to ten times higher compared to the stations SH2 and O9. The Secchi depth varied between the stations, but was only 15–30% of the mean water depth (Table 3). The stations GB19 and RB10 showed a positive correlation between chl *a* and TP (GB19, *r* = 0.532, *p* < 0.001, *n* = 51; RB10, *r* = 0.315, *p* < 0.05, *n* = 40). In DB2, there was no correlation between TP and chl *a* (*p* > 0.05, *n* = 44), but a high variability between TP and chl *a* (Fig. 2b). Two µmol L^−1^ P, for example, can correlate with chl *a* (= biomass) between 10 and 80 µg L^−1^. The highest TP and chl *a* concentrations were measured in the innermost CWB (Fig. 2c). The long-term medians for chl *a* were at least twice as high compared to other inner costal water bodies (DB16—101, KHM—70, P42—80 µg L^−1^). The median TP concentrations in these system were two to three times higher (DB16—4, KHM—6.5, P42—4.8 µmol L^−1^) compared to other stations. The Secchi depths at those stations were lowest during summer with around 10% of the mean water depth (Table 3). Surprisingly, the station with the highest chl *a* (DB16) had the lowest TP and DIP concentrations of the innermost stations and chl *a* did not correlate with TP (*p* > 0.05, *n* = 45).

**Table 3:** Proportion of Bacillariophyceae (%B), Chlorophyta (%G) and Cyanobacteria (%C) (2007–2014) and Secchi depth (m) and Chlorophyll *a* concentration (µg L^−1^) (2000–2014) as median for all stations. Spring—March, April, May; Summer—June, July, August. O9—Arkonasee, SH2—Salzhaff, DB2—Grabow, GB19—Greifswalder Bodden, RB10—Großer Jasmunder Bodden, KHM—Kleines Haff, P42—Peenestrom, DB16—Saaler Bodden

Station	Spring					Summer				
	%B	%G	%C	Secchi	Chl *a*	%B	%G	%C	Secchi	Chl *a*
O9	0	3.2	23.7	5	2.2	0	8.0	38.0	4.75	2
SH2	14.9	0.3	16.6	4.1	2.5	0	2.2	14.8	3.8	3.5
DB2	n.d.	n.d.	n.d.	0.5	40.4	n.d.	n.d.	n.d.	0.6	32.1
GB19	39.2	1.4	14.0	1.7	10.5	16.6	4.9	55.6	1.35	11.8
RB10	5.3	21.7	47.2	0.8	29.9	3.6	12.6	66.6	0.8	30.8
KHM	50.0	9.0	6.0	0.6	96.8	15.2	6.8	46.5	0.5	70.1
P42	33.7	16.1	31.8	0.65	68.3	14.7	10.7	64.9	0.5	80
DB16	8.8	11.0	73.2	0.25	117	4.1	5.2	89.2	0.25	101

Interestingly, DB16 and DB2 are part of the same lagoon system and showed both the same deviating chl *a*-to-TP ratios. Further, those stations had the lowest Secchi depth (DB16—0.2 m, DB2—0.6 m, on median), compared to the chl *a* concentration of other stations. The chl *a* values of the stations KHM and P42 correlated significantly with TP (KHM, *r* = 0.456, *p* < 0.005, *n* = 38; P42, *r* = 0.643, *p* < 0.001, *n* = 42).

### Effects of nutrient concentrations on phytoplankton composition

Cyanobacteria contributed half of the biomass during summer months at five stations (Table 3). Bacillariophyceae and Chlorophyta contributed only small amounts (up to 15%) of the total biovolume during summer months at all stations. However, at GB19, KHM and P42 Bacillariophyceae were dominant during springtime. Cyanobacterial dominance continued during autumn at the five stations GB19, RB10, KHM, P42 and DB16 (data not shown). Interestingly, there was a total dominance of cyanobacteria at the station DB16 during the complete sampling period (75–90%). The station DB2 was not sampled for phytoplankton by the LUNG, but species composition was identical to station DB16 (Schumann et al. 2008).

The less saline, innermost water bodies were dominated by cyanobacteria, but phytoplankton community composition was highly variable among all stations. Cyanobacteria were dominated by species of the genera *Anabaena*, *Aphanothece*, *Limnothrix*, *Merismopedia*, *Microcystis*, *Planktolyngbya*, *Snowella*, and *Synechococcus*. The biovolume of cyanobacteria correlated positively and significantly with TP concentrations at three of the seven sites (RB10, *r* = 0.3, *p* < 0.03, *n* = 52; KHM, *r* = 0.4, *p* < 0.005, *n* = 46; P42, *r* = 0.62, *p* < 0.001, *n* = 30). At the same time, cyanobacteria showed a negative correlation to the amount of TN at the two stations with lowest salinities (DB16, *r* = −0.28, *p* < 0.04, *n* = 58; KHM, *r* = −0.5, *p* < 0.001, *n* = 45). Bacillariophyceae correlated positively with TP at the station O9 (*r* = 0.315, *p* < 0.05, *n* = 61), but negatively at the stations KHM and P42 (KHM, *r* = −0.331, *p* < 0.03, *n* = 46; P42, *r* = −0.535, *p* < 0.002, *n* = 30). TN correlated also positively with Bacillariophyceae biovolume at four stations that represented the whole observed gradient for salinity (SH, *r* = 0.4, *p* < 0.03, *n* = 29; DB16, *r* = 0.44, *p* < 0.001, *n* = 58; KHM, *r* = 0.33, *p* < 0.03, *n* = 45; GB19, *r* = 0.29, *p* < 0.03, *n* = 55). The biovolume of Chlorophyta correlated negatively to TP only at the station KHM (*r* = −0.387, *p* < 0.01, *n* = 46). It was surprising that biovolume correlated with TP at RB10, KHM and P42, but not with chl *a*, even though chl *a* correlated with TP at those stations.

### Ecosystem response to high precipitation events

**Fig. 3:**
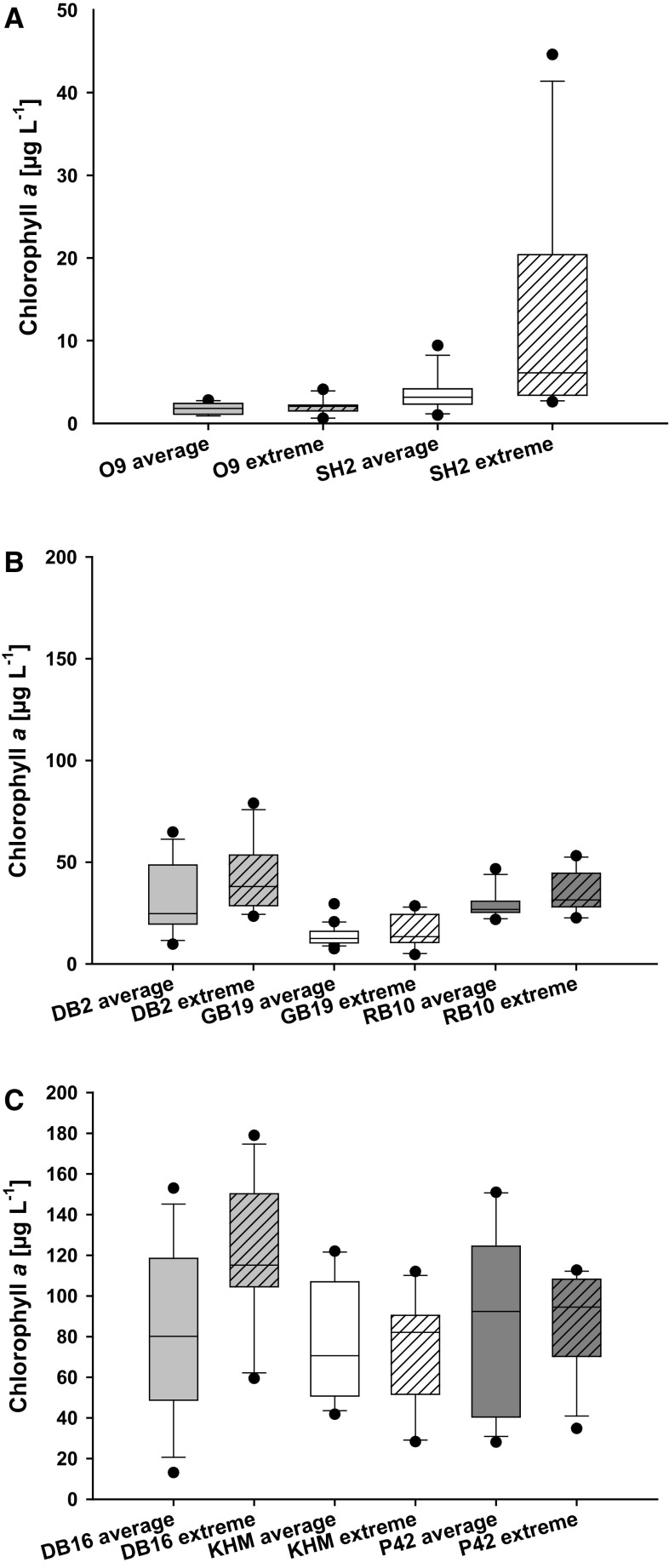
**a–c** Whisker-Box plots for chlorophyll *a* (µg L^−1^) in comparison of summer (June–August) with average precipitation and summer with extreme precipitation (extreme precipitation >150% the long-term average; years 2002, 2007, 2010, 2011). The stations were sorted regarding their chl *a* concentration, please note the different scalings. O9—Arkonasee, SH2—Salzhaff, DB2—Grabow, GB19—Greifswalder Bodden, RB10—Großer Jasmunder Bodden, KHM—Kleines Haff, P42—Peenestrom, DB16—Saaler Bodden

Extreme precipitation events were measured during the summer months of 2002 (1.6 times higher median), 2007 (1.9 time higher median), 2010 (1.5 times higher median), and 2011 (3.4 times higher median). Figure 3 shows how extreme precipitation can impact the chl *a* concentrations in CWB. Five out of 8 stations exhibited at least a 15% higher chl *a* concentration during summer months at above-average precipitation. This stimulating effect of higher precipitation on biomass was detectable for all stations regardless if localised at the outer coastal line or representing an inner water body.

**Fig. 4:**
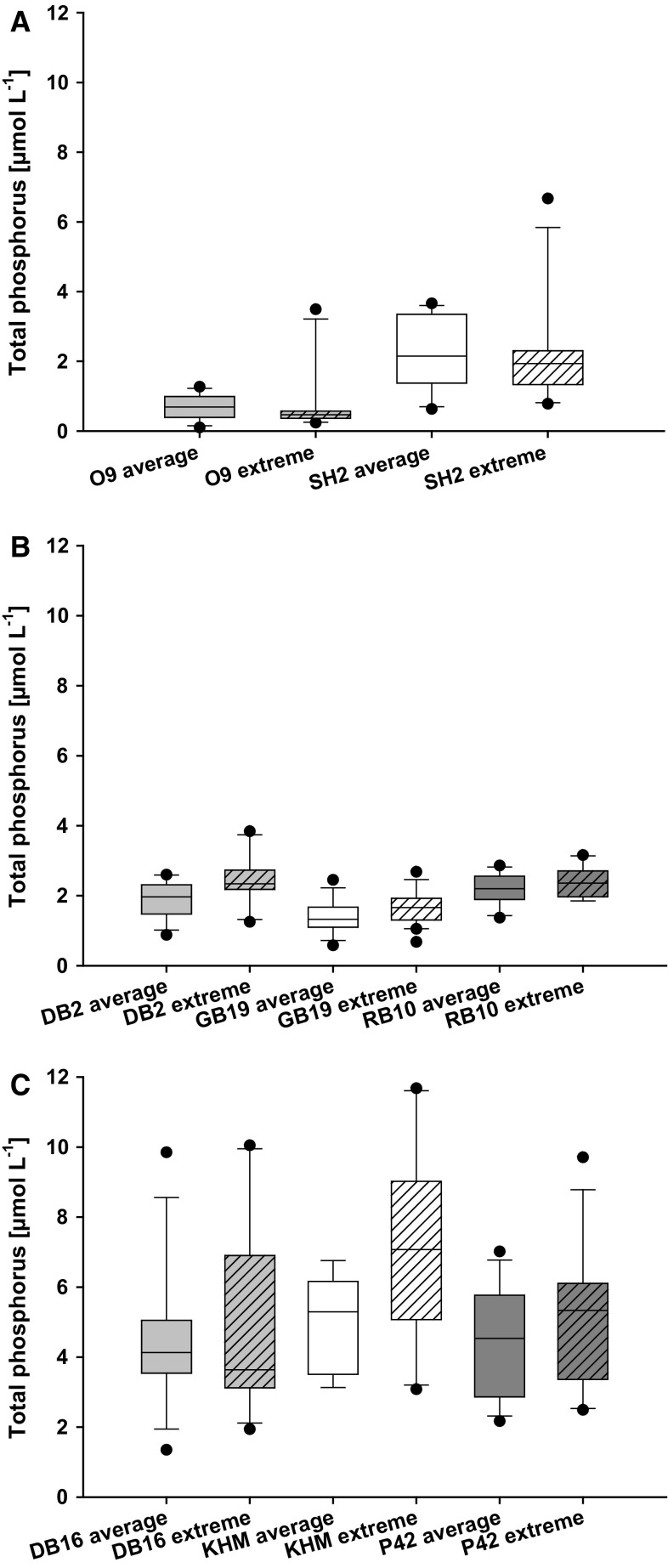
**a–c** Whisker-Box plots for total phosphorus (TP) (µmol L^−1^) in comparison of summer with average precipitation and summer with extreme precipitation (extreme precipitation >150% the long-term average, years 2002, 2007, 2010, 2011). The stations were sorted regarding their TP concentration. O9—Arkonasee, SH2—Salzhaff, DB2—Grabow, GB19—Greifswalder Bodden, RB10—Großer Jasmunder Bodden, KHM—Kleines Haff, P42—Peenestrom, DB16—Saaler Bodden

**Fig. 5:**
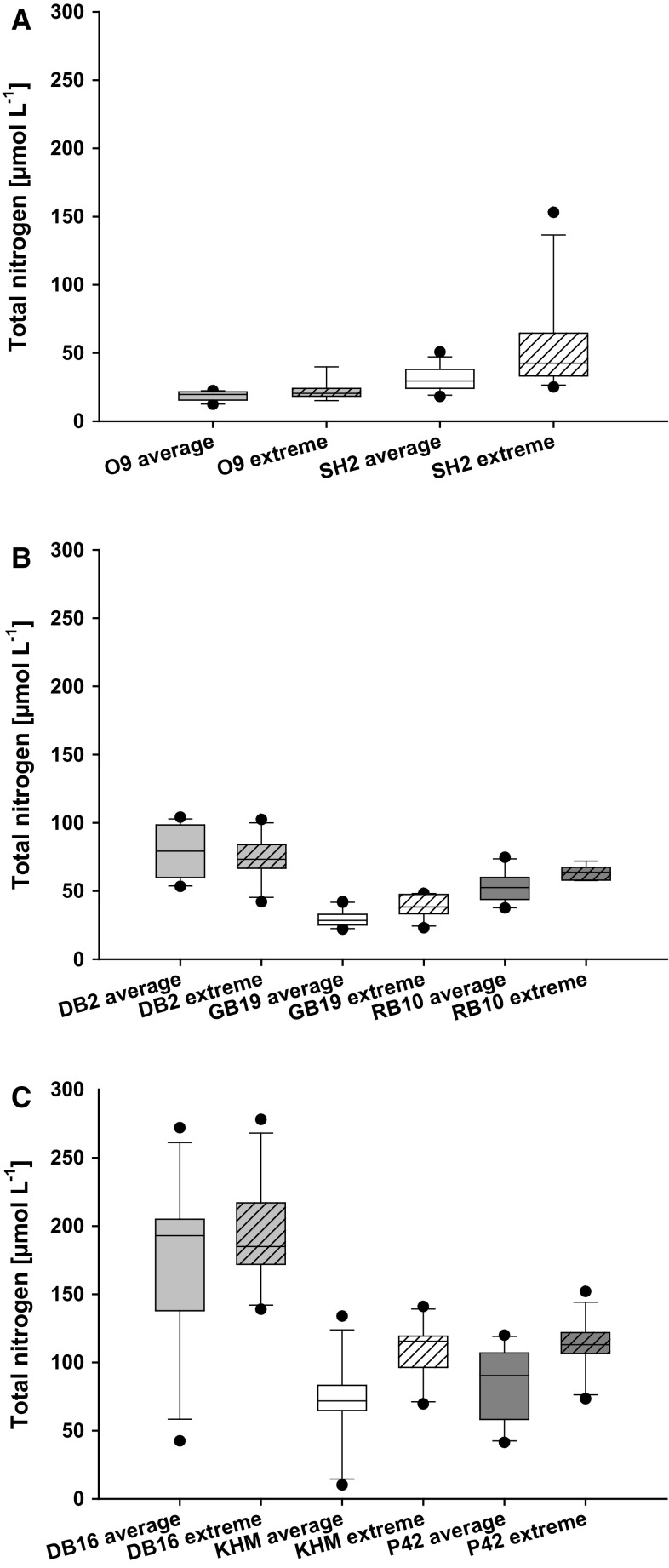
**a – c** Whisker-Box plots for total nitrogen (TN) (µmol L^−1^) in comparison of summer with average precipitation and summer with extreme precipitation (extreme precipitation >150% the long-term average, years 2002, 2007, 2010, 2011). The stations were sorted regarding their TN concentration. O9—Arkonasee, SH2—Salzhaff, DB2—Grabow, GB19—Greifswalder Bodden, RB10—Großer Jasmunder Bodden, KHM—Kleines Haff, P42—Peenestrom, DB16—Saaler Bodden

The data set revealed no immediate TP increase during years with higher precipitation, compared to years with average precipitation at the same stations (Fig. 4). The stations DB2, GB19, KHM and P42 had higher TP concentrations on median, but only DB2 and P42 differed significantly (t-test, DB2, *p* < 0.05, *n* = 12; P42, *p* < 0.05, *n* = 12). Furthermore, the DIP concentrations were only above median at station KHM during extreme years, the same station that had always detectable DIP concentrations. All other stations showed their highest median DIP concentrations (at least two times higher) during summer months with average precipitation (data not shown). However, it was possible to link the events of high precipitation to elevated TN concentrations (Fig. 5). Five out of eight stations showed higher TN concentrations during years with high precipitation (*t* test, GB19, *p* < 0.002, *n* = 12; SH2, *p* < 0.03, *n* = 12; KHM, *p* < 0.01, *n* = 12; P42, *p* < 0.01, *n* = 12; RB10, *p* < 0.01, *n* = 12). The DIN concentration was never significantly elevated in years with high precipitation compared to average years.

## Discussion

### Phytoplankton community responses on nutrient availability

The current TP values were two to six times higher than the desired target values for the German inner CWB of the Baltic Sea during summer. DIP concentrations were at 5 out of 8 stations in a determinable range (>0.05 µmol L^−1^), but DIP was always low (on median ≤0.1 µmol L^−1^) at stations with highest cyanobacteria proportion (DB2, DB16, RB10). However, elevated DIP concentrations were frequently observed during summer at all stations. The chl *a* values found in this work were two to twelve times higher at the same TP levels compared to e.g. Danish coastal waters (Kronvang et al. 2005) or brackish lagoon systems in Australia (Cook and Holland 2012). Chl *a* values in those studies were probably lower, because of elevated dilution with open sea water as indicated by salinity. The DIP and TP values observed in this study indicate that P might only be the limiting factor at the observed stations during winter and spring. The high DIN values promote the formation of high phytoplankton blooms during spring, as indicated by the positive correlation of Bacillariophyceae, as spring-dominant phytoplankton, with TN and overall decreasing nutrient concentrations. The TN:TP as well as the DIN:DIP ratio during spring point to a P limitation. The high DIN:DIP ratios are in contrast to those of the Baltic Proper, where even during winter times DIN:DIP ratios of 16:1 are measured with low DIN concentrations (~ 8 µmol L^−1^ DIN) (Walve and Larsson 2007). The most important observation during seasonal development of phytoplankton is the fact that DIN becomes unavailable in summer whereas DIP still occurs, which might explain the dominance of cyanobacteria. A N-limitation was also described, for example, for most days in Danish coastal waters (Kronvang et al. 2005), Australian lagoons (Cook and Holland 2012) and the Thames estuary (Nedwell et al. 2002) during decade-long water quality surveys. Consequently, the high chl *a* concentrations are mainly controlled by N rather than by P, even though the TN:TP ratios indicate P limitation. A cause for that can be the cyanobacteria dominance. Finkel et al. (2010) showed in their review that cyanobacteria can have higher N:P ratios (up to 50:1) compared to other taxa. These findings indicate that the TN:TP ratio found in this work does not necessarily indicate a P limitation. Therefore, it is so important to evaluate carefully the phytoplankton composition. Albrecht et al. (2017) showed in their most recent work that the pure morphological identification of cyanobacteria as used by environmental agencies is not possible. These authors determined, for example, *Aphanothece* and *Synechococcus* morphologically at the inner coastal water body stations DB2, DB16 and RB10. However, the genetic determination contained mainly members of the *Cyanobium* clade, which may be misidentified morphologically due to its great morphological plasticity. *Cyanobium* is currently described to lack N-fixation genes (Scanlan et al. 2009). On the other hand, the N-fixation genes are polyphyletic and show a patchy distribution in the cyanobacteria phylogeny, i.e. not only the heterocystous species, but also many non-heterocystous taxa possess these genes (Latysheva et al. 2012). Therefore, it is at present impossible to evaluate whether N-limitation, as indicated by DIN:DIP, favours N-fixing cyanobacterial species, and whether there is a limitation at all. The concentrations of easily bioavailable dissolved organic N and P have to be considered to address this question. However, both parameters are currently not monitored.

The discrepancy between phytoplankton biomass and nutrients can be attributed to resilience mechanisms. The adaptation capabilities of cyanobacteria to low P levels need to be discussed since they were the dominating group at some inner coastal waters. Cyanobacteria are usually very small (<10 µm) and thus have a high cell surface-to-volume ratio. This ratio supports small-sized cells in term of competitive nutrient uptake (e.g. Friebele et al. 1978). Cyanobacteria are also capable to luxury consume P and store it as polyphosphate (e.g. Ritchie et al. 2001). This luxury consumption can explain the discrepancy of high TP values at moderate chl *a* concentrations, but not vice versa. Additionally, they are capable to cause higher turbidity per unit P and even adapt to the self-induced light limitation suppressing other phytoplankton species (Scheffer et al. 1994), which would explain high chl *a* concentrations at lower TP concentrations found in this study. These authors characterised the described data as hysteresis mechanism on the dominance of cyanobacteria in shallow lakes, which is in agreement with the results in the present study. Stations (i.e. DB2, DB16) with 75–90% cyanobacteria dominance had the lowest Secchi depth at same (DB16) or even half (DB2) the chl *a* concentration compared to other analysed stations (i.e. KHM, P42, see Table 3). The described resilience was already postulated by Sas (1989) for lakes. The luxury P uptake and intracellular P accumulation is especially important in dynamic aquatic systems like CWB (Aubriot et al. 2011), where nutrient supply typically occurs as seasonally fluctuating pulses. The adaptations for light limitation would explain the high chl *a* concentrations found in this work, but not the DIP concentrations that should have been luxury consumed. These findings lead to the conclusion that phytoplankton might be P saturated and shows a severe N-limitation during summer.

The described increased phytoplankton growth during high precipitation events might be explained by elevated nutrient input through the catchment area. This work exhibited that during years with above-average precipitation the water bodies were affected seasonally. An effect on phytoplankton was often measured, although the data set revealed no consistently higher TP concentration during those rainy summer months (vegetation periods). An elevated nutrient input during storm events (Pinckney et al. 2001) and humid years (Jordan et al. 2003) was described for other estuaries and even for tile drains within the catchment areas of German coastal zones (Zimmer et al. 2016). However, not only bioavailable P but also N is transported into coastal systems during extreme weather events and elevating the DIN:DIP ratio (up to 60:1 during storm events) (Rees et al. 2009). The elevated chl *a* concentrations during extreme years could be rather explained by the impact of N than by P. This assumption could also explain why higher DIP concentrations occurred during years with average precipitation, compared to years with higher precipitation. The enhanced demand for DIP during growth with simultaneous DIN loading may explain the low DIP concentrations during extreme years. All these findings agree with Howarth and Marino (2006) who postulated in their review that N is the eutrophication causing agent in most coastal marine systems, but primary production can only be reduced with a coupled N- and P-reduction.

### Implementation of phytoplankton resilience in coastal management

Schernewski et al. (2015) argued that the reduction aims for P are too high for the German Baltic coast. They used a model to calculate the chl *a* concentrations in the time around 1880 and nowadays. These authors concluded that CWB should have a higher reference value for chl *a* concentrations, therefore the P input can be higher into the coastal systems. However, this argumentation does not include the resilience factors during re-mesotrophication. The described resilience mechanisms and effects of stochastic events support the hypothesis of a hysteretic process. Duarte et al. (2009) described the problems with current management policies that they rule out the higher baseline or background of nutrients in the system and its adjacent land. The inflow of N by point sources was not reduced to the same extent as P into the German coastal waters (Nausch et al. 2011). In fact, the P reductions by point sources were during 1986–1990 around 60% and additional 6% during 2004–2008. The N reduction was only 13% for the same period of time (Nausch et al. 2011). Nowadays, both nutrients N and P access German surface water mainly by non-point sources (up to 80% of all inputs) (BMUB and BMEL 2016). These results indicate that both, N and P, are relevant for the ecology of the analysed coastal waters. However, DIP is not limiting and can be used very efficiently by the dominating phytoplankton composition. The amount of P bound in agriculturally used soils ranges in Germany between 34 and 192 mg kg^−1^ soil, depending on the type of use (no fertilisation, organic farming, animal manure) (Schick et al. 2013). The P accumulation in German soils is still positive with 1.8 kg ha^−1^ a^−1^, but already lower as in neighbouring countries like Denmark (10 kg ha^−1^ a^−1^) or Poland (7.4 kg ha^−1^ a^−1^) (van Dijk et al. 2016). The accumulated P is the potentially available amount that can be released during storm events and flush into the aquatic system. Therefore, a further reduction of the still accumulated P is necessary, before balancing the P input to soils. Other methods to reduce P loss from soils can be, for example, improved fertilisation timing, or enhanced soil mixing to prevent P stratification in soils (Sharpley et al. 2001; Sharpley 2003).

The following question arises: What happens when the P concentrations are reduced further in the catchment area of inner coastal waters? It is possible that the most adapted phytoplankton species to low P levels, like cyanobacteria, become more and more dominating. A stronger reduction of N could also lead to higher abundances of cyanobacteria, particularly those capable of N-fixation, with their disadvantages of toxin production and increasing turbidity (Scheffer et al. 1994; Anderson et al. 2008). This possible development leads to the question, if the “good ecological state” is achievable with nutrient reduction only. Additional counteractions have to be analysed and discussed.

Biomanipulation of aquatic ecosystems is such a possibility to support re-mesotrophication efforts. The methods range from submerged macrophyte planting to food web alterations. Submerged macrophytes can reduce the turbidity within an aquatic ecosystem (e.g. Kufel and Kufel 2002), compete with phytoplankton for nutrients (Ozimek et al. 1990) and simultaneously act as a refuge for zooplankton (e.g. Beklioglu and Moss 1996). Planting of submerged macrophytes is a promising method to recreate lost habitats and this has been successfully documented for other areas worldwide (Fonseca et al. 1994; van Keulen et al. 2003). The planting stabilises the sediment and reduces the turbidity by resuspended particles. Competition of macrophytes for nutrients with phytoplankton is possible. However, macrophytes tend to use nutrients rather from sediment pore water when available, than from the water column (Granéli and Solander 1988). Food web alterations include the removal of planktivorous fish (e.g. Jeppesen et al. 2007), or artificial stock piscivorous fish into aquatic ecosystems (Søndergaard et al. 1997). The major aim is to reduce grazing pressure on zooplankton. High zooplankton densities control phytoplankton abundance, leading to higher penetration depth of light and hence better conditions for macrophytes. However, all these methods have been mainly applied in shallow eutrophic freshwater lakes. It remains unclear, if these methods can be applied in CWB.

## Conclusion

Within the EU, the most challenging efforts have to be made during the next decades to reduce the human impact on aquatic systems. Many coastal water systems have already developed an alternative stable state after decades of eutrophication with specific phytoplankton species compositions and reduced, but still too high nutrient fluxes. It will need new ecological and management approaches to change these system-specific stable states with their intrinsic resilience mechanisms and finally reach a “tipping point” to return the CWB into a mesotrophic state. However, it might also be possible that some of these coastal water systems will never reach a “good ecological state”. Those systems will remain in their current state, simply because it is not possible, or cost-efficient to reduce the nutrient concentrations below the desired values. Stochastic non-manageable effects, like above-average precipitation can setback previous efforts. Future research efforts have to deal with dynamic CWB, including their exchange between adjacent freshwater and marine systems. More fundamental information is needed to understand and to manipulate re-mesotrophication processes and resilience mechanisms to effectively counteract eutrophication. Such studies have to include food web interactions and nutrient cycling. But the first action should always focus on the reduction or elimination of diffusive and point sources for nutrients in the catchment area, before restoration can take place in the CWB.
